# Effect of short and long moderate-intensity exercises in modifying cardiometabolic markers in sedentary Kenyans aged 50 years and above

**DOI:** 10.1136/bmjsem-2017-000316

**Published:** 2018-04-25

**Authors:** Karani Magutah, Rebecca Meiring, Nilesh B Patel, Kihumbu Thairu

**Affiliations:** 1 Department of Medical Physiology, University of Nairobi, Nairobi, Kenya; 2 Department of Medical Physiology, Moi University, Eldoret, Kenya; 3 Exercise Physiology Laboratory, Faculty of Health Sciences, School of Physiology, University of the Witwatersrand, Johannesburg, South Africa

**Keywords:** exercise physiology, glucose, lipids, non-communicable disease, physical fitness

## Abstract

**Objectives:**

We compared effects of shorter moderate-intensity exercise time (<10 min bouts) on cardiometabolic parameters with the current recommendations among elderly adults.

**Methods:**

Fifty-three sedentary individuals aged ≥50 years were divided into exercise groups^1^: male and^2^ female short-duration bouts (M_S_ and F_S_, respectively), and^3^ male and^4^ female long-duration bouts (M_L_ and F_L_, respectively). Short-duration bouts consisted three 5–10 min moderate-intensity jogging sessions daily, and long-duration bouts consisted 30–60 min sessions 3–5 days weekly. Cumulative exercise times were equivalent. Physical activity (PA) was measured by log and activity monitors. Fasting venous blood at baseline and 8 weekly intervals was used for blood chemistry.

**Results:**

After 24 weeks, M_S_ and F_S_ with total cholesterol (TC) of >5.2 mmol/L and >5.3 mmol/L decreased from 22.2% to 14.8% and from 30.9% to 11.5%, respectively. For M_L_, this decreased from 25.9% to 3.7%, while F_L_ had 0% change. In M_S_ and M_L_, TC/high-density lipoproteins (HDLs) of >5.0 mmol/L dropped from 22.2% to 7.4% and from 22.2% to 15.4%, respectively. In F_S_ and F_L_, TC/HDL of >4.5 mmol/L declined from 19.2% to 7.7% and from 19.2% to 3.8%, respectively. M_S_ and M_L_ with fasting blood glucose of ≥5.5 mmol/L declined from 40.7% to 11.1% and from 33.3% to 3.7%, respectively. Similarly, it declined from 46.2% to 0% and 42.3% to 11.5% for F_S_ and F_L_, respectively. There were no differences in the changes between regimes throughout the study.

**Conclusion:**

Bouts lasting <10 min per session are as good as those lasting;≥30 min in improving cardiometabolic profiles of sedentary adults aged ≥50 years.

**What are the new findings?**

- Majority of sedentary Kenyans aged above 50 years have metabolic health risks.
- Moderate-intensity exercise offered in <10 min bouts has similar effects to longer bouts in modifying cardio-metabolic markers in sedentary adults.
- Regardless of the sex, accumulated short bouts of moderate-intensity exercise that last less than 10 min have beneficial health effects.

## Introduction

The current recommendation for weekly duration of moderate-intensity PA for health benefit in adults is a minimum of 150 min, traditionally achieved in 3–5 sessions of 30–60 min per week, or accumulated through bouts that last more than 10 min.^1^ In some developed economies, 51%–79% of adults do not meet this weekly PA recommendation.^1 2^ In Eldoret, Kenya, where the current study was performed, more than 82% of elderly adults do not achieve recommended levels of exercise.^3^ A lack of time due to other responsibilities partly contributes to lack of adherence to this PA recommendation.^1 4–6^ To improve adherence, it has been recommended that more experimental studies on the intensity, frequency and duration of exercise be undertaken and that would have a more global appeal on the benefits of exercise^1 7–11^ and, in particular, studies that lead to more appealing exercise regimes for older adults.

In the 1990s, suggestions were made that short exercise bouts were as effective as single continuous sessions of similar intensities provided that the cumulative weekly exercise time is equal.^12^ However, evidence for this suggestion remains inconclusive as existing guidelines still focus on either single continuous sessions or bouts that last at the very least 10 min. This has thus failed to mitigate the poor exercise adherence observed among the older populace.^13^ Studies on the efficacy of even shorter exercise regimes that last less than 10 min remain scarce, leading the WHO to highlight the need for further studies on effectiveness of short-duration exercise.^1^

The cardiometabolic health benefits received from exercise for the elderly is clear, with a strong linkage to improved cardiorespiratory fitness (CRF),^14–18^ but the specific type of exercise regime that shows higher adherence yet yields similar cardiometabolic benefits needs to be determined. Several studies^12 19 20^ have reported benefits of accumulated shorter exercise bouts. The lack of longer term follow-up in these studies has raised the question of the long-term benefits^21–23^ the short bouts may accrue. Therefore, in this study, cardiometabolic markers were compared for 24 weeks between sedentary elderly adults undertaking long-duration exercise bouts lasting ≥30 min and those performing short-duration exercise bouts that lasted <10 min.

## Materials and methods

### Participants

Healthy sedentary males (n=27) and females (n=26) aged ≥50 years were recruited from Eldoret, Kenya, following a local print advertisement. Sedentariness was defined as exercise/activity amounting to <600 metabolic equivalent minutes, using the WHO Global Physical Activity Questionnaire (GPAQ). A physical examination to rule out existing health problems was performed on all volunteers on their verbal declaration they were in good health. Individuals with cardiorespiratory disease or other physical ailments/injuries were excluded. Also excluded were participants on β-blockers and participants with diabetes on therapy. Signed informed consent was obtained from all participants.

### Protocol

A biodemographics questionnaire on exercise history was completed for each participant following which they were randomly allocated into four groups^1^: male short-duration bouts of exercise (M_S_),^2^ female short-duration bouts of exercise (F_S_),^3^ male long-duration bouts of exercise (M_L_) and female long-duration bouts of exercise (F_L_). In the short-duration bouts of exercise groups, the participants engaged in three sessions of 5–10 min of moderate-intensity jogging daily. In the long-duration bouts of exercise groups, participants engaged in 30–60 min jogging sessions for 3–5 days each week. Data from the participants were collected and analysed at the recruitment stage (September 2016) and then at 8-week intervals for a period of 24 weeks after the start of the exercise intervention to culminate in May 2017. Participants kept an exercise log, and objective verification of adherence to the exercise regime was done weekly using Polar Wearlink ActITrainer Accelerometers (Actigraph, Pensacola, Florida, USA), and activity monitors were fastened onto participants on select days. The data, metabolic equivalent minutes achieved from the activity monitors, were analysed weekly to ascertain that participants in the different exercise regimes had comparable cumulative exercise time. Since this was only available for select days and used to primarily verify adherence, exercise intensity was monitored using WHO GPAQ. For clinical analysis, 4 mL of venous blood was collected from an average of two participants daily following an overnight 12 hours’ fast, usually between 06:00 and 08:00. Two drops of fleshly drawn blood were immediately used to measure blood glucose using Freestyle Optium Glucometer (Abbott, Oxfordshire, UK). For lipid profiles, centrifugation of blood was done within an hour of collection following which the serum was frozen and kept at −12°C, awaiting to run accumulated weekly samples together. This analysis into total cholesterols (TCs), high-density lipoproteins (HDLs), low-density lipoproteins (LDLs) and triglycerides (TGs) was done using Cobas Integra 400 Plus (Roche, Germany) at the Academic Model Providing Access to Healthcare Reference Laboratories, a certified clinical laboratory run by Indiana University/Moi University partnership.

### Analysis

Data analysis was done at univariate, bivariate and multivariate levels based on sex of participant and their exercise regime. Paired t-tests were used for intragender differences of metabolic markers following the different exercise regimes. The outcome variables were further analysed at the multivariate level by performing multiple analysis of variance for the dependent variables of lipid profiles and blood glucose and, further, linear regressions controlling for sexgroup performed. For comparison across the four phases of the experiment, repeated measures analysis of variance was conducted for equality of means of these blood chemistry results. Analysis was done using STATA V.13; p value was set at ≤0.05.

## Results

The mean age of the males (n=27) was 55.5±3.0 years and 53.9±3.0 years for the females (n=26). The proportion of participants who had completed tertiary education was 88.4% among the male and 70.4% among the female. Ninety-one per cent of participants were in white-collar jobs. All (100%) male and female participants in the M_S_ (n=14) and F_S_ (n=13) group adhered to their 24-week exercise regime, compared with 61.5% of the M_L_ (n=14) and 76.9% of F_L_ (n=13). The weekly cumulative exercise minutes was similar for M_S_ and M_L_ (161.8±7.2 vs 162.56±6.1, respectively) and also for F_S_ and F_L_ (158.3±3.6 vs 156.05±2.7, respectively). Specific baseline characteristics for participants from the four groups are presented in table 1.

**Table 1:** Baseline characteristics of participants in the four groups

	M_S_	M_L_	F_S_	F_L_
Age (years)	55.0±5.6	55.2±3.0	53.9±2.6	53.9±3.5
BMI (kg/m^2)^	25.8±4.0	28.6±4.8	33.3±4.8	32.0±5.4
Waist/height ratio	0.52±0.07	0.56±0.08	0.61±0.05	0.57±0.08
Waist/hip ratio	0.93±0.06	0.96±0.07	0.82±0.10	0.84±0.09
Fat %	20.7±7.3	24.9±7.9	39.8±3.6	37.4±5.1
Resting systolic pressure (mm Hg)	138.9±17.4	140.8±22.1	133.7±13.2	137.6±23.8
Resting diastolic pressure (mm Hg)	82.1±11.3	83.7±8.7	83.7±10.6	84.2±8.1
Resting heart rate (b/m)	73.9±9.5	76.8±7.7	79.8±12.0	71.8±8.4

Data presented as mean±SD.

BMI (kg/m^2^), basal metabolic index in kilograms per meter squared; b/m, beats per minute; F_L_, long bouts female; F_S_, short bouts female; M_L_, long bouts male; M_S_, short bouts male.

Mean fasting blood glucose (FBG) of all participants at the start of the study was 5.92±1.4 mmol/L (males) and 6.23±0.74 mmol/L (females). The percentage of males and females with FBG >5.5 mmol/L was 40.7% (M_S_ group), 33.3% (M_L_ group), 46.2% (F_S_ group) and 42.3% (F_L_ group). In 22.2% (M_S_), 25.9% (M_L_), 30.8% (F_S_) and 11.5% (F_L_), baseline TC was above the cut-off of >5.2 mmol/L for males and >5.3 mmol/L for females. HDLs below 0.9 mmol/L was found in 18.5% (M_S_), 11.1% (M_L_), 7.7% (F_S_) and 15.4% (F_L_). TC/HDL ratio of >5.0 mmol/L and>4.5 mmol/L, the cut-offs for males and females, respectively, was found in 44.4% of the males (22.2% each in M_S_ (mean=6.58±1.21) and M_L_ (mean=6.63±1.29)) and 38.5% of the females (19.2% each in F_S_ (mean=5.38±0.92) and F_L_ (mean=5.42±0.59)). LDL/HDL ratio >3.5 was found in 14.8% M_S_ group (mean=4.07±0.53) and in 18.5% M_L_ group (mean=4.79±1). LDL/HDL ratio >3.0 was found in 23.1% F_S_ group (mean=3.53±0.27) and 11.1% F_L_ group (mean=3.32±0.43).

**Figure 1:**
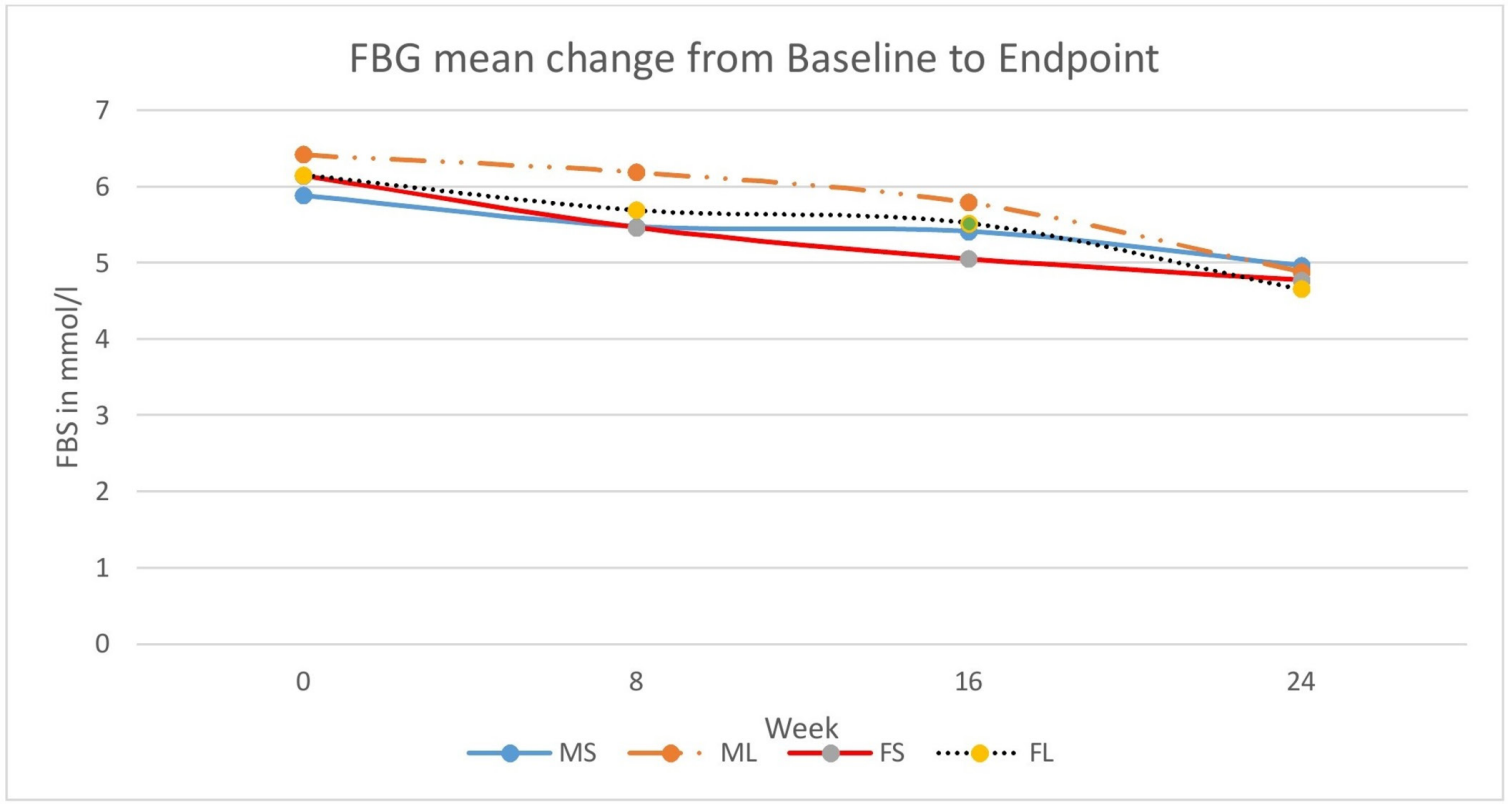
Mean change in fasting blood glucose (FBG) between week 0 and week 24. For each exercise regime, FB dropped over the 24-week period in both sexes. FBG, fasting blood glucose; FL, long bouts female; FS, short bouts female; ML, long bouts male; MS, short bouts male.

**Figure 2:**
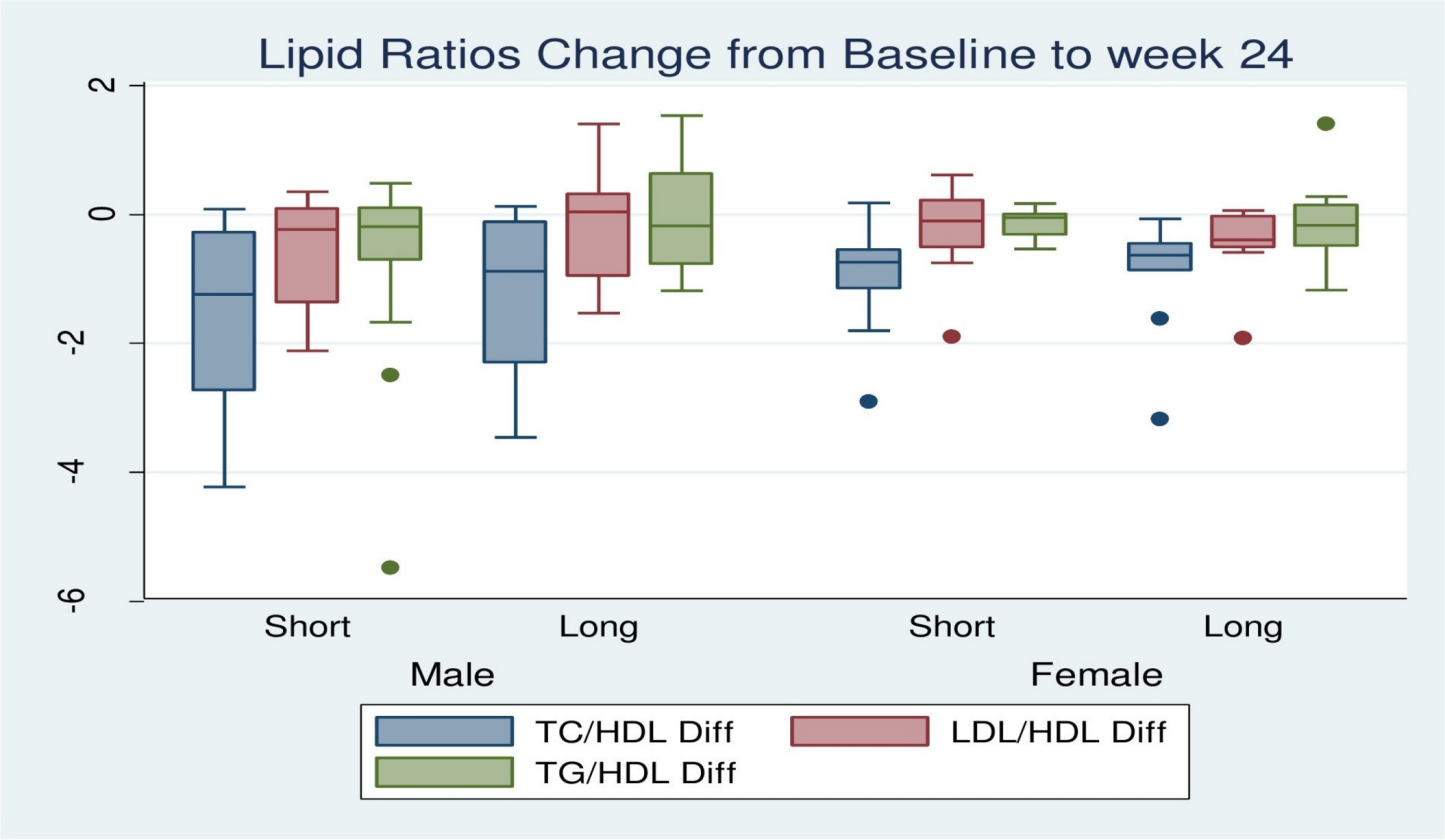
Mean change in lipid ratios between week 0 and week 24. No differences were observed in the change in lipid ratios in 24 weeks for the different exercise regimes in each either sex. HDL, high-density lipoprotein; LDL, low-density lipoprotein; TC, total cholesterol; TG, triglycerides.

At the end of the 24-week period, male participants with FBG of ≥5.5 mmol/L in both the M_S_ and M_L_ groups decreased by 29.6%. The percentage of female participants in F_S_ group with FBG of ≥5.5 mmol/L dropped from 46.2% to 0, while in F_L_ group, it decreased by 30.8%. Mean glucose values for all the groups also dropped, but there was no significant gender difference between different exercise regimes (figure 1). All M_S_ and F_S_ who had baseline HDL of <0.9 mmol/L reached values of ≥0.9 mmol/L, the recommended lower cut-off. There was no percentage change in the subjects in M_L_ group with HDL <0.9 mmol/L. Seventy-five per cent of F_L_ group reached HDL ≥0.9 mmol/L. For TC in the M_S_ group, participants with >5.2 mmol/L fell from 22.2% to 14.8%, with mean value decreasing from 6.4±1.38 mmol/L to 5.48±0.41 mmol/L. In the M_L_ group, the reduction was from 25.9% to 3.7%, with mean TC decreasing from 6.09±0.58 mmol/L to 5.76 mmol/L. Similarly, in F_S_ group, participants with TC >5.3 mmol/L decreased from 30.8% to 11.5% with the mean TC decreasing from 6.75±1.46 to 5.72±0.15 mmol/L. In F_L_ group, there was no change in number of participants with TC >5.3 mmol/L, although their mean values decreased from 7.78±1.79 mmol/L to 5.76±0.44 mmol/L. For the TC/HDL ratio, in the M_S_ group participants with ratios >5.0 decreased from 22.2% to 7.4% (mean decreased from 6.58±1.21 to 5.59±0.36), and in F_S_ group, those with the ratio >4.5 decreased from 19.2% to 7.7% (mean decreased from 5.38±0.92 to 5.14±0.03). In the M_L_ group, the decrease was from 22.2% to 15.4% (mean change from 6.63±1.29 to 5.66±0.5), and in F_L_ group, the decrease was from 19.2% to 3.8% (mean change from 5.42±0.59 to 5.18±0). Percentage of M_S_ group with LDL/HDL >3.5 mmol/L was halved to 7.4% with mean LDL/HDL decreasing to 3.61±0.14. In the M_L_ group, the decrease was from 18.5% to 11.1% with mean LDL/HDL decreasing to 4.65±0.67 mmol/L. In F_S_ group, participants with LDL/HDL ratio >3.0 decreased from 23.1% to 15.4% (mean 3.49±0.55) and to 3.8% from 11.1% (mean 3.49±0) in the F_L_ group. Mean changes for the lipid ratios are provided in figure 2.

In summary, except for TC in females where the short bouts appeared superior to the long bouts, there was no difference in actual value change between the two bouts for the various cardiometabolic parameters over the 24-week period as shown in table 2. Table 3 is linear regression outcomes controlling for gender in the difference of the means above.

**Table 2:** Mean cardiometabolic values over 24 weeks

Variable	Group	Baseline	Week 8	Week 16	Week 24	Mean Δ(week 24 to week 0)	P values
Male							
TC	Short	5.4±1.3	4.8±1.2	4.8±0.9	4.7±0.8	−0.68±1.3	0.99
	Long	5.0±0.8	4.7±1.2	4.8±1.0	4.3±0.7	−0.68±0.5	
HDL	Short	1.3±0.5	1.3±0.4	1.5±0.4	1.6±0.4	0.30±0.4	0.55
	Long	1.0±0.3	1.0±0.3	1.1±0.4	1.2±0.5	0.21±0.3	
LDL	Short	3.1±0.9	3.1±0.8	2.9±0.9	3.1±0.7	0.04±0.7	0.81
	Long	2.8±1.2	3.2±1.5	2.9±1.2	2.9±1.3	0.13±0.9	
TG	Short	1.8±1.3	1.6±0.9	1.7±0.8	1.6±0.5	−0.23±1.2	0.42
	Long	1.9±0.9	2.4±1.8	2.3±0.9	2.1±1.1	0.16±0.8	
TC/HDL	Short	4.8±1.9	4.0±1.5	3.6±1.6	3.2±1.3	−1.52±1.4	0.65
	Long	5.4±1.9	5.1±1.9	5.0±1.7	4.1±1.8	−1.24±1.3	
LDL/HDL	Short	2.8±1.2	2.6±1.0	2.3±1.3	2.2±0.9	−0.59±0.9	0.30
	Long	3.0±1.5	3.2±1.8	2.9±1.4	2.9±1.7	−0.16±1.0	
TG/HDL	Short	1.8±2.0	1.5±1.1	1.4±1.2	1.1±0.6	−0.75±1.6	0.26
	Long	2.2±1.6	3.1±3.7	2.6±2.0	2.2±1.9	−0.04±0.9	
Female							
TC	Short	5.8±1.7	4.9±0.5	5.0±0.8	4.9±0.5	−0.91±1.4	0.01
	Long	4.0±1.0	4.8±0.8	4.7±0.6	4.5±1.2	−0.51±0.9	
HDL	Short	1.3±0.3	1.2±0.2	1.4±0.3	1.4±0.3	0.12±0.4	0.07
	Long	1.0±0.3	1.2±0.2	1.3±0.2	1.4±0.3	0.41±0.3	
LDL	Short	3.6±0.9	3.5±0.7	3.1±0.9	3.5±0.8	−0.03±0.6	0.11
	Long	2.6±0.9	3.4±0.7	2.8±1.0	3.1±1.0	0.45±0.8	
TG	Short	1.3±0.5	1.2±0.4	1.1±0.4	1.3±0.6	−0.04±0.4	0.21
	Long	1.0±0.3	1.2±0.4	1.1±0.3	1.1±0.3	0.19±0.5	
TC/HDL	Short	4.5±1.0	4.1±0.8	3.7±0.8	3.6±0.9	−0.88±0.8	1
	Long	4.2±0.7	4.0±0.7	3.7±0.6	3.3±0.9	−0.88±0.9	
LDL/HDL	Short	2.8±0.8	2.9±0.8	2.3±0.7	2.5±0.8	−0.23±0.7	0.45
	Long	2.7±0.5	2.8±0.7	2.2±0.8	2.2±0.7	−0.43±0.6	
TG/HDL	Short	1.1±0.5	1.0±0.4	0.8±0.3	0.9±0.5	−0.13±0.2	0.81
	Long	1.2±0.6	1.0±0.4	0.9±0.4	0.9±0.6	−0.18±0.7	

Values are means±SD in millimoles per litre of blood. P values represent if the difference in mean change between bouts is statistically significant.

Δ, change; HDL, high-density lipoproteins; LDL, low-density lipoproteins; TC, total cholesterol; TG, triglycerides.

**Table 3:** Linear regressions for cardiometabolic variables (controlling for sex)

Mean change, long	Coefficient	SE	P>|t|	95% Cl
FBG	−0.36	0.25	0.15	−0.87 to 0.14
TC	0.75	0.36	0.05	0.15 to 1.48
HDL	0.11	0.11	0.33	−0.11 to 0.33
LDL	0.29	0.23	0.20	−0.17 to 0.75
TG	0.30	0.24	0.22	−0.18 to 0.78
TC/HDL	0.13	0.34	0.70	−0.56 to 0.82
LDL/HDL	0.10	0.24	0.68	−0.38 to 0.58
TG/HDL	0.31	0.32	0.33	−0.33 to 0.95

FBG, fasting blood glucose; HDL, high-density lipoproteins; LDL, low-density lipoproteins; TC, total cholesterol; TG, triglycerides.

## Discussion

### Baseline outcomes

Lipid profiling is crucial in CRF assessment among healthy individuals and those with metabolic syndrome. At the start of the study, nearly half of the males and females had TC above their reference cut-offs (>5.2 and >5.3 mmol/L, respectively). A third of the males and females had unfavourable levels of both LDL and HDL. Additionally, one-fifth and one-tenth of males and females had high TG levels. For TC/HDL associated with poor CRF and cardiovascular disease,^24–26^ about half from both sexes had values higher than cut-off value. Similarly, a third of males and females had unfavourably high LDL/HDL, and about one-tenth had TG/HDL consistent with cardiometabolic risks. Current cut-offs used for TG/HDL^25^ are from non-black populations, and these may be different in other populations. Combination of various lipids and their ratios showed in the study participants a large proportion being at substantial metabolic risk at the start of the study. For FBG, participants had prediabetic to diabetic mean similar to those found in studies on other African populations of comparable ages,^27 28^ and mean values for females were higher than their age-matched males. Results from urban Nigerians and rural Kenyans^29 30^ differ from the baseline measurement found in this study, probably because we studied a relatively older population. Thus, a majority of this urban population had metabolic health risks at recruitment, a finding similar to that recently found among rural Kenyans.^30^

### Absolute lipid profile changes

After 24 weeks of prescribed moderate-intensity exercise, both males and females showed improved metabolic profiles. Nearly half of the M_S_ group with unfavourable TC at the start reached values associated with better CRF. Furthermore, mean TC in these males decreased as did the mean for those who still did not reach the recommended ranges. The finding was similar among M_L_ group, suggesting the two exercise regimes produced comparable affect. This M_L_ group also had a reduced percentage that remained with values above recommended cut-off and a decline in mean values for the whole group and, importantly, those who did not attain TC values <5.2 mmol/L as well. The two exercise regimes produced similar changes in the mean TC at the end of the study period. In females, except for the decrease in mean TC being higher in F_S_ group, which suggests short-bout exercise could have better outcome compared with the currently recommended exercise regime, no other differences were observed in TC. Also, two-thirds of females with high baseline TC in F_S_ group reached the recommended range at the end of the study; none from the F_L_ group did. The two exercise regimes, however, had comparable decrease in the overall TC means, even among participants who were unable to achieve the reference cut-off. When baseline and end-point values were compared, mean TC change was similar in the two regimes. Thus, based on TC alone, no demonstrable difference could be shown between the exercise regimes for either sex. No study that we are aware of has described this exercise effect in individuals of comparable age and setting and followed for similar time-length as our study.

Males and females in both exercise regimes had an increase in HDL. This finding is consistent with other studies that show exercise affects HDL.^31 32^ Specifically, males and females in short-bout exercise regime whose baseline HDL was <0.9 mmol/L (18.5% and 7.7%, respectively) decreased to 0. In the F_L_ group, those with HDL <0.9 mmol/L, also decreased from 15.4% to 3.8%, and there was no change in the M_L_ group. What our study adds and that could be a beneficial interventional approach is that the short sessions appeared more beneficial in improving HDL levels when compared with the traditional regimes. For LDL and TG, the change was marginal. Males and females on long-bout exercise regime had slight rise in both LDL and TG, but there was slight improvement in the short-bout exercise participants.

### Effect on lipid ratios

For lipid ratios, two-thirds of M_S_ and F_S_ group showed reduced TC/HDL (<5.0 and <4.5, respectively). In M_L_, it was a quarter, and in F_L_, it was four-fifths. Previously, it has been suggested that intermittent exercise regimes may actually be more beneficial than current exercise regimes in regulation of attributes such as blood pressure and maximal oxygen consumption.^33^ What our study adds is that short exercise regimes are also beneficial in improving TC/HDL among males. Our results, however, show mixed outcomes since for the females, long-bout exercise regime was marginally superior in improving the TC/HDL ratio. This improvement in females differs from Quinn *et al*,^33^ who found that shorter exercise regimes provide better outcomes, although their study excluded a comparison of TC/HDL. Furtherore, our study period was twice as long (24 weeks vs 12 weeks), and each of our exercise sessions took half the number of minutes (7.5 min vs 15 min) adopted by Quinn *et al*,^33^ which could have contributed to the difference in our findings. Furthermore, since the long-bout exercise regime was less effective compared with the short-bout exercise regime in lowering TC in females, the difference in results could be due to the long-bout exercise raising HDL marginally higher thereby reducing the TC/HDL ratio. The decrease in mean TC/HDL in males and females in the two exercise regimes who did not achieve recommended levels was similar. With regard to TC/HDL ratio, the effect of the two exercise regimes could not be differentiated, and the apparent dissimilarity in the effect among females was not supported when controlling for sex. Thus, the effect on TC/HDL by the two exercise regimes was similar in both males and female.

LDL/HDL had lower change in rate throughout the study period. However, the overall trend was similar in both sexes from the two exercise regimes. Half of M_S_ group with baseline values of >3.5 showed lower ratios. Slightly less than half of M_L_ regime participants also showed LDL/HDL ≤3.5. There was no difference between the groups in overall mean change of LDL/HDL for the duration of the study. The exercises regimes also had comparable mean values in those not reaching the recommended LDL/HDL ranges. Fifty per cent of F_S_ group that started with high values of LDL/HDL reached the recommended threshold, compared with about two-thirds in the F_L_ group. Thus, shorter exercise regime was more favourable in LDL/HDL regulation in males, although females still attained satisfactory results since drop in LDL/HDL for traditional group was not statistically superior to that in experimental group. Based on TC/HDL and LDL/HDL, it is apparent the two exercise regimes had similar reduction of cardiovascular disease risk. This could also address the recent finding, which our baseline data also support, that majority of Kenyans aged above 50 years are at cardiovascular disease risk based on combination of various lipid ratios.^30^ Regardless of gender, all would thus benefit similarly, whether from short or long sessions of exercise. Overall, comparison of these ratios suggest that no difference exists in reduction of metabolic syndrome risk in individuals in either of these exercise regimes.

### Blood glucose effects

FBG levels decreased for all groups. In M_S_ group, participants with FBG >5.5 mmol/L decreased from 40.7% to 11.1%, while in the M_L_ from 33.3% to 3.7. Absolute FBG mean change, however, was similar between the groups, and participants retaining prediabetic-to-diabetic values were less in M_S_ and F_S_ than in the M_L_ and F_L_ groups. In the F_S_ group, all participants with prediabetic-to-diabetic baseline FBG levels (46.2%) achieved normal levels after the 24 weeks. In the F_L_, this was from 42.3% to 11.5%. The mean change in absolute FBG values between baseline and end-point were similar for F_S_ and F_L_ groups. Regression analysis controlling for gender showed no significant difference between the short-duration and long-duration bouts of exercise in FBG mean change. The view that longer exercise sessions among the males and the shorter regime for the females were superior based on percentage change (alone) therefore failed to hold further. This, coupled with the observation of no difference in mean change for FBG after 24 weeks for both sexes from the two different exercise regimes, supports our hypothesis that these exercise regimes have similar effects. Thus, shorter/intermittent exercise sessions lasting <10 min each regulate elevated FBG to the same extent as the longer traditional sessions lasting >30 min per session, as long as the cumulative exercise times are similar.

### Limitations

The design of the protocol may have caused some limitations. Our sample was recruited through print advertisement, which self-selection may have favoured those able to read, compromising inference to the general elderly population. Furthermore, the design could not allow for blinding, depended on subjective health assessment at recruitment with only a physical examination the only form of objective verification, and did not consider lifestyle behaviours such as smoking history and diet, which could have affected health status of participants. These potential limitations may have confounded our results and affected generalisation.

## Conclusion

An exercise regime of accumulated short bouts lasting <10 min shows improvement of cardiometabolic measurements among sedentary adults aged ≥50 years comparable with the currently advocated longer sessions that last >30 min each, as long as the intensity and cumulative exercise times are similar.
